# Habitual vs Non-Habitual Manual Actions: An ERP Study on Overt Movement Execution

**DOI:** 10.1371/journal.pone.0093116

**Published:** 2014-04-01

**Authors:** Jan Westerholz, Thomas Schack, Christoph Schütz, Dirk Koester

**Affiliations:** 1 Center of Excellence Cognitive Interaction Technology (CITEC), Bielefeld, Germany; 2 Neurocognition and Action - Research Group, Faculty of Psychology and Sports Science, University of Bielefeld, Bielefeld, Germany; 3 Research Institute for Cognition and Robotics (CoR-Lab), Bielefeld, Germany; Centre national de la recherche scientifique, France

## Abstract

This study explored the neurophysiological mechanisms underlying the planning and execution of an overt goal-related handle rotation task. More specifically, we studied the neural basis of motor actions concerning the influence of the grasp choice. The aim of the present study was to differentiate cerebral activity between grips executed in a habitual and a non-habitual mode, and between specified and free grip choices. To our knowledge, this is the first study to differentiate cerebral activity underlying overt goal-related actions executed with a focus on the habitual mode. In a handle rotation task, participants had to use thumb-toward (habitual) or thumb-away (non-habitual) grips to rotate a handle to a given target position. Reaction and reach times were shorter for the habitual compared to the non-habitual mode indicating that the habitual mode requires less cognitive processing effort than the non-habitual mode. Neural processes for action execution (measured by event-related potentials (ERPs)) differed between habitual and non-habitual conditions. We found differential activity between habitual and non-habitual conditions in left and right frontal areas from −600 to 200 ms time-locked to reaching the target position. No differential neural activity could be traced for the specification of the grip. The results suggested that the frontal negativity reflected increased difficulty in movement precision control in the non-habitual mode compared to the habitual mode during the homing in phase of grasp and rotation actions.

## Introduction

We seamlessly and effortlessly pick up and manipulate objects in our everyday life. We usually do so with the consequences of our behavior in mind, indicating the cognitive effort underlying motor planning and control. Planning processes before action execution have been shown in a study by Rosenbaum et al. [1]. Participants did not seem to strive for a comfortable grip (overhand) and to avoid an uncomfortable grip (underhand) when grasping a bar. Apparently, participants preferred a comfortable hand posture at the *end* of the movement when placing the bar onto a target position. Rosenbaum et al. [1] suggested that participants anticipated their future hand postures and called this effect the end-state comfort effect, as the participants showed a preference for final comfort over initial comfort. In the experiment, participants had to take hold of a bar lying on a pair of cradles. There was a target position on both sides of the cradles, one to the left and one to the right. Participants had to grab the bar and bring either the right or left end of the bar to the right or left target position. If the right end of the bar had to be placed on one of the two targets, participants grasped it with an overhand grip. If the left end of the bar had to be placed on one of the two targets, participants grasped it with an underhand grip. Further experiments found sequential effects for motor planning that further emphasize the role of mental representations for motor control [2], [3], [4], [5].

The question why people seem to prefer comfortable end states has not been answered yet. It might be that ending comfortably provides better control or more precision at the end of the movement, or when this is needed [6]. A habitual system would be another explanation for grasp choices [7]. The habitual system favors movements that were rewarding in the past and, therefore, grasps that people habitually use for object manipulation. Most studies in this area focused on bar-transport tasks with a vertical or horizontal orientation of the bar, while there are only few experiments covering more orientations. Following the work of Rosenbaum et al. [8] we investigated a more fine-grained version of the bar-transport task. Surprisingly, although cognitive aspects demonstrated by the end-state comfort effect were frequently highlighted, neurophysiological studies for the overt execution of goal-related grasps are hard to find. The aim of this study was to investigate the neural mechanisms underlying the overt execution of goal-related actions with a focus on habitual vs non-habitual grasps.

One possible explanation for the end-state comfort effect is the precision hypotheses. Precision requirements are oftentimes higher at the end of the movement. Ending in a comfortable posture allows for greater precision and faster movements because faster movements are possible at the middle of the range of motion [9], [10]. A wider range of motion would also lead to greater control at the end of the movement. Further evidence for this hypothesis comes from another study by Rosenbaum, Vaughan, Jorgensen, Barnes and Stewart [8]. They used a handle connected to a disk which was turned clock-like from a starting position to a target position. The handle was constructed in a way that allowed subjects to grasp it at its rotational axis. A pointer at one end of the handle indicated its orientation. Eight numbers around the perimeter were used as possible target positions. The experimenter announced a target number. Then the subjects had to take hold of the handle and turn the disk until it showed in the direction of the target. The disk had low friction and had to be carefully brought to the target position. All required rotations included 180 degrees. Again, subjects showed the end-state comfort effect. That is, the probability of grasping the handle with the thumb towards the pointer was related to the pointer's final position. The minimum of the probability, for participants performing the task with their right hand, was near the 4 o'clock position, which was presumably the most awkward posture. For participants performing the task with their left hand, the minimum probability was near 7 o'clock, again, the presumably most awkward posture. The authors hypothesized that participants ended the task in a comfortable posture because this ensured precise task completion.

In line with the precision hypothesis, Rosenbaum et al. [9] showed that the end-state comfort effect can be eliminated when the precision requirements at the end of the movement are eliminated. The previous experimental setup [8] was modified so that no more precision was needed to bring the disk in the target position. The disk locked in automatically when it reached the target position. Half of the subjects did not show the end-state comfort effect. Rosenbaum et al. [9] suggested that the subjects who showed the end-state comfort effect did so only because they overestimated the precision requirements of the task. It seemed that participants' initial grasp choices were influenced by the anticipated precision or control needed at the end of the task. Further findings indicating that movements are not planned towards end-state comfort but rather towards a comfortable posture at the moment, when control is needed, have been reported by Hughes et al. [11] and Künzell et al. [12]. Hughes et al. [11] varied the precision demands at the beginning and end of a bar transport task and observed initial state comfort for 50% of their participants. In the experiment of Künzell et al. [12], participants had to grasp a bar and move it through obstacles of varying size at the beginning and end of the movement. Künzell et al. suggested that movements were planned for optimal control during the movement part that demands the highest precision.

In addition to the end-state comfort effect, Rosenbaum et al. [8] observed a preference for grasping the handle with the thumb towards the pointer. Participants did not perform the same handle rotations, for example the rotation from position 1 to position 5 and the rotation from position 5 to position 1, with the same movements. Instead, they showed a tendency to grasp the handle with the thumb towards rather than away from the pointer. The authors called this effect, which they observed also in another experiment [13], the thumb-towards bias. They suggested that attentional factors explain the effect, as the thumb and index finger are more strongly associated with attention than the little finger.

A contrasting explanation for the thumb-towards bias was proposed by Herbort and Butz [7]. They interpreted the grip position as a habitual bias, as most tools used in everyday life are grasped with the thumb toward the functional end of the tool. Künzell et al. [12] argued in favor of a habitual mode as long as no specific demands, like precision demands, require a cognitive-motor planning process. The aforementioned studies provided evidence that cognition and action are strongly interwoven. They indicated that people grasp objects depending on what they intend to do with them. Grasp selection seems to be influenced by the action goal and also by a habitual mode.

In line with behavioral studies, neurophysiological findings suggested that voluntary actions were planned and executed with their intended goal in mind. In a recent review Waszak et al. [14] described that the medial frontal cortex seems to play a crucial role in linking actions to their predicted effects. The brain also seems to pre-activate the representation of the predicted action effect during action selection [14].

In an fMRI study, van Elk et al. [15], investigated the planning processes of object-directed actions using a motor imagery task. Participants had to imagine how to execute actions with familiar and unfamiliar objects based on goal- or grip-related information. They observed increased activation in parietal areas for unfamiliar objects and explain this with the involvement of parietal areas in motor imagery, which might take more effort for unfamiliar actions. For familiar objects, they observed increased activation in anterior prefrontal cortex and suggested that there is a stronger goal-representation for actions with familiar objects compared to unfamiliar ones.

There is neurophysiological evidence for different control mechanisms underlying goal-directed actions, which depend on the goal-posture. Most existing studies in this field focused on button presses, mental simulation, and action preparation intervals, but few studies investigated the planning and execution of overt complex actions by means of ERPs.

One example for such an ERP study is the work by van Schie and Bekkering [16], who investigated neural mechanisms underlying immediate and final action goals for precision grips. They used a grasp and transport task and instructed either the grasp participants had to use (immediate goal) or the end position of the transport (final goal). Although participants executed the same overt movement in both conditions, Van Schie and Bekkering observed different ERPs for immediate and final action goals. The immediate goal was accompanied by a parieto-occipital slow wave, while the final goal was accompanied by a slow wave over left frontal regions. The authors suggested that the enhanced activation found in posterior parts for the immediate goal indicate this area's involvement in the prehension of the object, while the enhanced activation found in anterior parts for the final goal might indicate frontal involvement in the planning and control of sequential behavior. This research showed that different neural mechanisms control the action depending on whether the emphasis is on the immediate or final goal of an action sequence.

Westerholz et al. [17] found a similar effect for the planning and execution of goal-related power grips, but with a distinct temporal pattern. They differentiated cerebral activity for the same action executed with an emphasis on initial vs. final parts of the movement sequence. In a grasp and transportation task, the relative emphasis was either on the grip (the immediate goal) or on the target location (the final goal). ERPs differed between immediate and final goal-cued conditions, suggesting different means of operation dependent on goal-relatedness. Differences occurred from −600 to −200 ms time-locked to movement end over right frontal areas. In accordance with previous findings [16], [18], [19], the results suggested that a parieto-frontal network is of crucial importance for grasp planning and execution.

A further experiment by Westerholz et al. (unpublished data) indicated that ERPs differ between self-regulated and instructed conditions in a bar transport task, but only when the action effect is manipulated, suggesting different ways of operation dependent on goal-relatedness. Bozzacchi et al. [19] suggested that action preparation is affected by the meaning of the action and by the awareness of being able to perform it. They performed an EEG study and compared the preparation phases of grasping for cup, impossible grasping of a cup (where the grasp was mechanically hindered) and reaching for a cup. In a related experiment, Bozzacchi et al. [20] recorded ERPs for a virtual grasp, a real grasp and a key-press. They suggested once more that action preparation is affected by the meaning of the action and that this is true for virtual actions as well.

The aforementioned studies served as a starting point for the present study. Participants executed a handle rotation task inspired by Rosenbaum et al. [8]. They had to grasp a handle and rotate it to a specified target position. The grip they used to take hold of the handle was either free choice or specified by the instruction. The specified instructions included two different types of grip. The grip was either a thumb-toward grip or a thumb-away grip. In the thumb-toward condition participants had to grasp the handle with the thumb or the base of the thumb toward the end of the handle that had to be rotated to the target position. In the thumb-away condition participants had to grasp the handle with the thumb or the base of the thumb pointed away from the end of the handle that had to be brought to the target position. The thumb-toward condition represented the use of a habitual mode, as in everyday life tools are mostly used with the thumb towards the functional end of the tool [7]. Thus, the thumb-away condition represented the use of a non-habitual mode. The aim of the present study was twofold. First, we aimed to extend existing knowledge for the execution of free choice and specified choice goal-related rotation tasks to the neurophysiologic field. Second, we aimed to differentiate between different neural control processes for action execution determined by the habitual mode and, thus, provide a more detailed account for pre-specified goal-related actions.

Previous studies [16], [17] (Westerholz et al., unpublished data) found different time windows in the time range from −900 to 0 ms time-locked to grasping for a grasp and transport task. This time range is of special importance for action planning and execution, when the same goal related action was executed but planned differently. The same studies found the time range from −1100 to 200 ms time-locked to movement end to be of importance for action planning and execution. As we investigated the planning and execution of a related task, a goal related grasp and rotation task, we hypothesized that neurophysiological processes, underlying grasping, reflect action planning in this time range.

As mentioned above, several studies [16], [17] reported goal-related effects on motor control processes time-locked to grasping over parietal-occipital cortex. Based on these results, we predict differential cerebral activity for the habitual condition compared to the non-habitual condition over parietal occipital cortex time-locked to grasping. Those studies further reported goal-related effects time-locked to movement end over left and right frontal regions. Thus, we predict differential cerebral activity for the habitual condition compared to the non-habitual condition over left and right frontal regions time-locked to movement end. We predict no significant difference for the specified grip choice and free grip choice conditions, because the determination of the initial grip of an action sequence should have no major effect on the planning and execution of the whole action sequence.

We predicted that participants would show the end-state comfort effect in the free grip choice condition. Based on the results of Rosenbaum et al. [8], we expected the end-state comfort planning to be most activated for the biomechanically most difficult postures, especially uncomfortable end postures. That is, for right hand grips the end-state comfort effect would be strongest at a 4 o'clock end position and for left hand grips it would be strongest at an 8 o'clock end position. In addition to the end-state comfort effect, we predicted that participants would act according to the thumb-toward bias [8] in the free grip condition. That means, participants would show a tendency to grasp the handle with the thumb toward the end which has to be rotated to the target position.

We predicted reaction times, reach times, and transport times to be faster for the habitual condition compared to the non-habitual condition. The habitual preference might show up in reaction, reach, and rotation times in the specified grip choice condition, in faster times for the habitual condition compared to the non-habitual condition. Rosenbaum et al. [13] reported that, in general, participants reacted faster when they grasped a bar with the thumb towards a pointer than when they grasped away from it. The authors further suggested that reaching for the bar started before participants had finalized their handgrip decision, which must then have been completed while the hand was in motion. Other studies [16] have already reported faster times for habitual movements. Previous bar-transport experiments [17] (Westerholz et al., unpublished data) have shown that not only the reaction time, reflecting planning processes before movement onset [21], [22], but reach and transport times which represent online planning, motor implementation processes, and movement execution, were affected as well.

We predicted no significant difference for reaction times between the specified grip choice and free grip choice conditions, whereas we expected reach and rotation times to be faster for the free grip choice condition compared to the specified grip choice condition. Fleming et al. [23] differentiated free and instructed choices and found similar preparation levels for both conditions, thus we expected no significant differences for reaction times. However, due to habitual reasons we expected that less decision making will be necessary in the free grip choice compared to the specified grip choice condition. These processes might show up after action initiation, when the hand is already in motion [13], [17](Westerholz et al., unpublished data).

## Materials and Methods

### Participants

Twenty eight healthy volunteers (mean age 25.43 years; SD 3.6; 18 females) with no known neurological impairments and normal or corrected-to-normal vision participated in the study. All participants were right-handed, which was evaluated with the Edinburgh Handedness Inventory (mean handedness score: 97.5)[24]. All participants were compensated for their time with course credit or money. All participants provided written informed consent and the experimental procedure was approved by the ethics committee at Bielefeld University, and adhered to the ethical standards of the sixth revision of the Declaration of Helsinki.

### Design and setup

**Figure 1:**
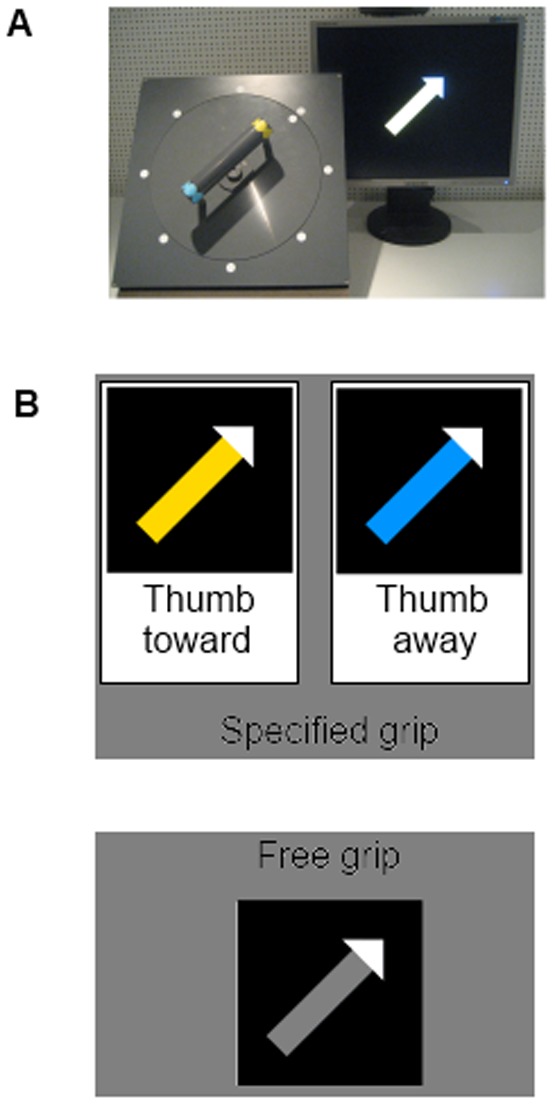
Task design. (A) Task setup showing the apparatus with the handle that had to be grasped with the thumb towards or away from the marker. Then it had to be rotated to a position indicated by the stimulus screen. (B) Possible stimuli for all conditions showing the grasp to use and the final orientation of the handle. Blue and yellow represent specified grips. A yellow arrow requires a grip with the thumb towards the yellow mark and thus towards the pointing direction. A blue arrow requires a grip with the thumb towards the blue mark and thus away from the pointing direction. A grey arrow indicates a free grip choice for the participant. The white arrow head points to the final orientation of the handle.

Participants executed a grasp and rotation task under three different conditions (Fig. 1). Instructions included specified or free-choice grip postures and a specified goal-position, where the rotation had to end. The three conditions were: 1. Specified grip posture with the thumb facing towards the end of the handle which had to be brought to a specified goal-position; 2. specified grip posture with the thumb facing away from the end of the handle which had to be brought to a specified goal-position; 3. free-choice grip posture of whether the thumb was facing towards or away from the end of the handle which had to be brought to a specified goal-position.

Participants were required to reach for a handle which was connected to a disk, grasp it with a power grip, and turn it to a goal position. A white marker was located on the disk, at one end of the handle. When the handle was rotated, it turned the disk and the white marker. Depending on the position of the white marker, it could point to one of eight equally spaced white markers that were located just beyond the perimeter of the disk. The end of the handle that was facing the white marker was marked yellow, while the end of the handle that was facing away from the white marker was marked blue. A start button was located in front of the apparatus with the handle.

In each trial, a picture stimulus was presented indicating the grip posture and goal location. First, the handle had to be grasped and turned from its initial position to the final goal location. Then, participants had to press the start button shortly. Afterwards, the disk automatically turned to the next start position.

The bar had to be rotated 180 degrees on 80% of all trials; these were the experimental trials. The remaining 20% of trials required varying degrees of rotation and were used as filler trials. Every start position of the handle was used for the same number of trials. The order of start positions was randomized. The picture stimuli consisted of arrows, showing the grip posture and goal location. The arrowhead was white and pointed to the goal location. The color of the arrow's shaft, which was either yellow, blue, or grey, indicated the grip posture. Yellow indicated a grip with the base of the thumb facing towards the yellow marked end of the handle and thus towards the white marker. Blue indicated a grip with the base of the thumb facing towards the blue marked end of the handle and thus away from the white marker. Grey indicated a free choice between the two possible grip postures. Stimuli for all conditions were shown in a randomized order.

### Procedure

Following electrode preparation, participants were seated comfortably in front of the table with the experimental setup. Participants received written instruction on the upcoming task. They were given information on how to grasp and turn the handle and were instructed to maintain stable posture and not to blink during trials. All questions they had concerning the instructions were answered.

The setup was calibrated to each participants' size to prevent expansive movements. The apparatus was positioned in front of the shoulder of the used arm and hand, such that participants could reach it comfortably with an extended arm. The start button was positioned in front of the apparatus, such that it could be reached with the hand comfortably. Participants were instructed to relax and not to tense up during the action. Picture stimuli were presented on a video monitor, which was located directly in front of the participant and laterally to the apparatus. Before the experiment started, participants performed short blocks of test trials until they performed the task correctly. These test blocks were also used to observe the EEG for obvious artifacts and were repeated until participants executed the task correctly in a relaxed state.

Each trial started when participants pressed the start button. First, a fixation cross for a randomized duration between 500 and 1500 ms was shown. Next, a picture stimulus was shown indicating the grip posture and the goal position of the handle. The stimulus remained on the screen until participants had reached the goal position. Participants were instructed to keep their gaze on the center of the screen throughout the movement. The next picture stimulus instructed participants to shortly press the start button. The disk then automatically turned to the next start position. Afterwards, a picture stimulus instructed the participants to press down the start button again, which started the next trial. The timing of all actions (hand lift, rotation start, rotation end) were registered. The experiment consisted of two blocks of 120 trials each. Participants used one hand for the first block and the other hand for the second block. They received instructions again for the second hand and also performed test trials until they performed the task correctly. Half of the participants performed the task with their right hand first, the other half performed the task with their left and first. Participants repeated tasks for each of the specified grip conditions 48 times (24 with their left hand, 24 with their right hand) and for the free choice grip condition 96 times (48 with their left hand, 48 with their right hand). The stimulus presentation was controlled by Presentation® software (version: 14.1, www.neuro-bs.com).In a post-experiment questionnaire, participants rated the difficulty of the task for each condition on a scale from 1 (easy) to 6 (difficult).

### Behavioral and electroencephalographic recordings

Behavioral recordings included the time points of lifting the hand off the start button, starting to turn the handle, and reaching the goal location. Micro switches were used to detect the exact moment they occurred. These events were recorded on the PC which was presenting the stimuli, as well as on the PC which was recording the EEG. Participants' performance was recorded with a video camera for later offline analysis.

EEG was recorded by a 64 channel amplifier (ANT). A WaveGuard EEG cap (ANT) with sixty-four Ag/AgCl electrodes was used. The electrodes of the cap were arranged according to the international 10-10 system (based on the 10-20 system)[25]. In order to detect ocular artifacts, EOG was recorded using four electrodes placed above and below the right eye and lateral to both eyes. During recording the data were average-referenced. The EEG was band-pass filtered (DC-138 Hz) and digitized at 512 Hz. The impedance of all electrodes was less than 5 kΩ.

### Data analysis

Video recordings were studied offline for performance errors. A trial was rated as containing an error when the participant used the wrong grip, changed the grip during the execution phase of the movement, or let go of the handle before the required goal position was reached. Trials with performance errors were excluded from the analyses. For correct trials, grasp behavior was documented.

Participants' average reaction, reach, and rotation times were subjected to a repeated measures ANOVA, to determine within-subject effects for grip type (specified grip posture thumb towards, specified grip posture thumb away, free grip posture). Based on the results of the ANOVA relevant conditions were then compared pair-wise by means of t-test.

For the comparison between different specified grip postures, behavioral analyses for reaction times (time from stimulus presentation to lifting of the hand), reach times (time from lifting the hand to rotation onset), and rotation time (time from rotation onset to rotation end) were each done separately. Averaged reaction, reach, and transport times were each subjected to a paired t-test to determine the influence of the condition (specified grip posture thumb towards, specified grip posture thumb away).

For the comparison between specified and free grip postures, behavioral analyses for reaction times, reach times, and rotation time were each done separately. Averaged reaction, reach, and transport times were each subjected to a paired t-test to determine the influence of the condition (specified grip posture, free grip posture).

Electrophysiological data were band-pass filtered offline from 0.1 to 30 Hz and re-referenced to the average mastoid electrodes. Response-locked analysis to grasping included the time interval from −2200–1200 ms. That means, epochs started −2200 ms before turning the handle from the start position and ended 1200 ms after the rotation started. Response-locked analysis to movement end included the time interval from −3200–300 ms. That means, started −3200 ms before reaching the target position and ended 300 ms after reaching it. Baseline correction was performed on the first 100 ms of each interval. Ocular artifacts were corrected using the correction procedure of Gratton et al. [26]. Artifact detection was done using a peak-to-peak moving window approach. Epochs containing peak-to-peak amplitudes above the threshold of ±50 μV within a 200 ms window were rejected. This window was moved over the whole epoch in 50 ms steps. 33% of the trials time-locked to grasping in the specified grip thumb toward condition, 34% in the specified grip thumb away condition, and 33% in the free grip posture condition were rejected due to artifacts. 34% of the trials time-locked to movement end in the specified grip thumb toward condition, 36% in the specified grip thumb away condition, and 34% in the free grip posture condition were rejected due to movement artifacts. For a comparison of thumb towards and thumb away conditions, the ERP was averaged separately for both experimental conditions. On average 30 trials per participant for the thumb toward condition and 29 trials for the thumb away condition entered analyses time-locked to grasping. On average 29 trials per participant for the thumb toward condition and 28 trials for the thumb away condition entered analyses time-locked to movement end. For a comparison of specified and free grip conditions, the data for specified thumb towards and specified thumb away grips were averaged together to form the specified grip condition, which was then compared to the free grip condition. On average 60 trials per participant for the free grip condition and 59 trials for the specified grip condition entered analyses time-locked to grasping. On average 60 trials per participant for the free grip condition and 58 trials for the specified grip condition entered analyses time-locked to movement end.

The EEG data were averaged for the left and right hand to avoid handedness effects. Hence, further observed lateral activity should not be evoked by handedness.

Mean amplitude analysis of the electrophysiological data included the factors *Condition* (thumb towards, thumb away; and separately specified grip, free grip), *Front-Back* (anterior, central, posterior) and *Left-Right* (left, middle, right). For the assessment of effects of scalp distribution, we differentiated between nine regions of interest (ROIs; anterior-left (AL): AF7, F7, F5, F3; anterior-middle (AM): F1, Fz, F2; anterior-right (AR): AF8, F8, F6, F4; central-left (CL): C3, C5, CP3, CP5; central-middle (CM): FCz, Cz, CPz; central-right (CR): C4, C6, CP4, CP6; posterior-left (PL): PO7, PO5, PO3, O1; posterior-middle (PM): Pz, POz, Oz; posterior-right (PR): PO8, PO6, PO4, O2). The Greenhouse-Geisser correction was applied when evaluating effects with more than one degree of freedom.

We analyzed the data in 100 ms step windows. To correct for false positives we combined these time windows into one, if three or more consecutive windows revealed significant 3-way interactions for Condition, Front-Back, and Left-Right, as well as for according t-tests [27]. In detail, we performed ANOVAs with the factors Condition (thumb towards, thumb away; and separately specified grip, free grip), Front-Back (anterior, central, posterior), and Left-Right (left, middle, right) for every single 100 ms time window of both epochs (time-locked to grasping and time-locked to movement end, incl. Greenhouse-Geisser correction where necessary). For time windows that revealed a significant 3-way interaction for Condition, Front-Back, and Left-Right, we performed t-tests for every ROI (see Tables S1 to S4). Only when three or more consecutive intervals reached the significance level (p<0.05), these intervals were combined, that is we averaged the amplitudes, to one time window. As a result, we analyzed the time window from −600 to 200 ms time-locked to movement end for thumb towards and thumb away conditions. Thus, the following statistics contain time windows, which consist of series of consecutive 100 ms steps that were found significant.

No significant effects were found for thumb towards and thumb away condition time-locked to grasping. No significant effects were found for specified and free grip conditions, neither time-locked to grasping, nor time-locked to movement end.

## Results

### Behavior & difficulty rating

Participants executed the task correctly in 96% of trials in both specified grip conditions. The remaining 4% of trials were rejected. Participants executed the task correctly in 97% of trials in the free grip condition. The remaining 3% of trials were rejected. They grasped towards yellow and thus towards the white marker in 81% of trials, and towards blue and thus away from the white marker in 16% of trials. For the probability of grasping with the thumb towards the marker for every final orientation see Table 1.

**Table 1:** Grasp behavior.

Final orientation	Probability of grasping thumb-toward (Left hand)	Probability of grasping thumb-toward (Right hand)
1	1.0	0.89
2	0.94	0.9
3	0.9	0.8
4	0.83	0.52
5	0.72	0.69
6	0.55	0.85
7	0.83	0.88
8	0.95	0.96

Probability of grasping with the thumb towards the marker in the free grasp condition for every final orientation for the left and right hand.

Participants rated the difficulty of the task in the specified grip thumb toward condition with 2.0, in the specified grip thumb away condition with 3.28, and in the free grip condition with 1.25 on a scale from 1 (easy) to 6 (difficult).

### Timing

A two-way ANOVA with the factors time (reaction time, reach time, rotation time) and grip type (specified grip thumb toward, specified grip thumb away, free grips) revealed a significant interaction for time and grip type, F(4, 108) = 58.8, p<0.001. Following the results of the ANOVA, we conducted three paired-samples t-tests to compare each of the reaction times, reach times, and rotation times in the corresponding conditions (Table 2).

**Table 2:** Average reaction, reach, rotation, and total execution time (in ms) and standard deviations (in brackets) for conditions that entered major analyses.

	Reaction time	Reach time	Rotation time	Total execution time
Habitual grip	651 (221)	979 (206)	1039 (228)	2669 (442)
Non-habitual grip	713 (293)	1311 (286)	1014 (194)	3039 (455)
Free grip	657 (198)	905 (193)	1002 (213)	2563 (395)
Specified grip	682 (256)	1145 (236)	1027 (199)	2853 (434)

For the specified grip condition data from the habitual grip and non-habitual grip condition were averaged together.

Reaction times were faster for specified grip thumb toward trials (651 ms) compared to specified grip thumb away trials (713 ms, t(27)  = −3.87, p<0.001). Reaction times were not significantly different for free grip trials (657 ms) compared to specified grip trials (682 ms, t(27)  = −1.73, p = 0.09).

Reach times were faster for specified grip thumb towards trials (979 ms) compared to specified grip thumb away trials (1311 ms, t(27)  = −11.62, p<0.001). Reach times were faster for free grip trials (905 ms) compared to specified grip trials (1145 ms, t(27)  = −10.3, p<0.001).

Rotation times were not significantly different for specified grip thumb towards trials (1039 ms) compared to specified grip thumb away trials (1014 ms, t(27)  = 0.9, p = 0.37). Rotation times were faster for free grip trials (1002 ms) compared to specified grip trials (1027 ms, t(27)  = −2.25, p = 0.03).

Execution of the whole action sequence was faster for specified grip thumb towards trials (2669 ms) compared to specified grip thumb away trials (3039 ms, t(27)  = −8.93, p<0.001). Execution of the whole action sequence was faster for free grip trials (2563 ms) compared to specified grip trials (2853 ms, t(27)  = −8.93, p<0.001).

### Electrophysiology

We conducted an ANOVA time-locked to movement end, which is the moment of reaching the goal position with the handle, with the factors Condition (thumb towards, thumb away), Front-Back (anterior, central, posterior), and Left-Right (left, middle, right).

**Figure 2:**
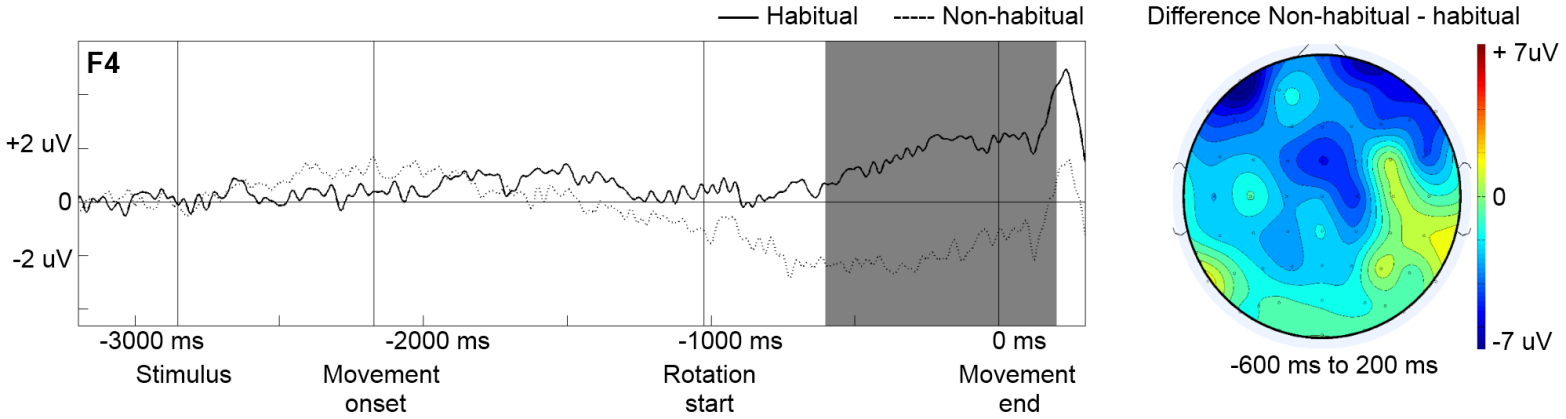
Slow wave brain potentials time-locked to movement end at electrode F4. (Left) Grand averaged ERPs recorded at electrode F4, time-locked to movement end, for the habitual (thumb toward) condition (solid) and non-habitual (thumb away) condition (dashed). The labels ‘Stimulus,’ ‘Movement onset,’ and ‘Rotation start’ mark the average time points of these events. (Right) Topography of the difference wave in the −600 to 200 ms time interval around movement end (indicated by the left grey selection) for the non-habitual condition minus the habitual condition.

The ANOVA for −600 to 200 ms revealed a significant 3-way interaction for Condition, Front-Back, and Left-Right, F(4, 108)  = 3.84, p = 0.01. The 3-way interaction meant that the ERP amplitude differences between the thumb toward and the thumb away condition was different in magnitude for the various combinations of the factors Front-Back and Left-Right. The significant interaction permitted the separate comparisons of the thumb towards and the thumb away conditions in the various regions-of-interest. We performed a t-test for every ROI to determine if there was a significant difference based on Condition and in which ROI this difference was present. A significant negativity for the thumb away condition compared to the thumb toward condition was present in the AL-ROI, t(27)  = 2.29, p = 0.03. A significant negativity for the thumb away condition compared to the thumb toward condition was present in the AR-ROI, t(27)  = 2.16, p = 0.04 (see Fig. 2). No significant effects were found for the remaining ROIs.

## Discussion

This study explored the neurophysiological mechanisms underlying the planning and execution of an overt goal-related handle rotation task. More specifically, we studied the neural basis of motor actions concerning the influence of the grasp choice. The aim of the present study was to differentiate cerebral activity between habitual and non-habitual grips, and between specified and free grip choices. In a handle rotation task, participants had to use thumb-toward (habitual) or thumb-away (non-habitual) grips to rotate a handle to a given target position. As predicted, the neural processes for action execution (measured by ERPs) differed between habitual and non-habitual conditions. We found differential activity between habitual and non-habitual conditions in left and right frontal areas from −600 to 200 ms time-locked to reaching the target position. However, no significant difference between both conditions appeared in analyses time-locked to grasping. In addition, we found no differential activity between free grip choice and specified grip choice conditions. The results indicated that the homing in phase of habitual and non-habitual actions were controlled by different neural processes which depend on the control requirements of the action. The results can be seen in line with the theory that anticipatory grasp choices are influenced by the demands of the task [6] and by habitual factors [7].

Participants executed the task correctly in 96% of trials in the habitual condition, 96% of trials in the non-habitual condition, and, hence, in 96% of trials in the specified grip condition, and in 97% of trials in the free grip choice condition. While this may have indicated that task difficulty did not differ between cueing conditions, participants rated the difficulty of the task in the non-habitual condition with 3.28, in the habitual condition with 2, and in the free grip choice condition with 1.25 on a scale from 1 (easy) to 6 (difficult). Thus, participants rated the non-habitual condition the most difficult. This confirmed our assumption that a thumb away grip was an uncommon grip, which our participants do not use habitually. However, the rating for the non-habitual condition provided a value near the middle of the scale between easy and difficult indicating that it was still unproblematic to execute the task.

In the free grip choice condition, participants showed a strong tendency to act according to the thumb-toward bias. They took hold of the handle with the thumb towards the pointer more often than away from the pointer for all target positions. The thumb-toward bias was much stronger than reported by Rosenbaum et al. [8] and, therefore, stronger than we expected. An explanation for this discrepancy could have been the kind of stimuli used to instruct the task. Our stimuli consisted of an arrow with a white head pointing to the target position. This kind of visual stimuli might have drawn participants' attention more to the pointer than did the auditory stimuli used by Rosenbaum et al. [8]. Thus, the stronger thumb-towards bias found here could be explained with attentional factors [13].

Due to the strong thumb-toward bias, the end-state comfort effect was not as pronounced as expected. Participants showed a tendency to act according to the effect. Their tendency to grasp the handle with the thumb-toward the pointer was lowest for target position 6 for left hand movements and target position 4 for right hand movements. This was in line with the results reported by Rosenbaum et al. [8]. They found the lowest probability for thumb-toward grasps for the same target positions and suggested that a thumb-away grasp for these positions would ensure a more comfortable end posture and thus more precision and control for the homing in phase of the movement. In addition to the explanation offered above, the results for the end-state comfort effect could have been influenced by the participants' perceived precision needed near the end of the turning movement. The stimulus presentation on the video monitor changed and the task was registered as complete, when the target position was first reached. That means, it was not necessary to accurately end on the target position to complete the task, but rotating the pointer through the target position would have been sufficient. Participants could have realized this during the experiment and, accordingly, could have ignored the precision demands of ending on target. However, none of the participants reported using such a strategy in the post experimental questionnaire. Offline analyses of the video footage did not support the explanation either, participants seemed to act as accurate as possible.

Reaction times (from stimulus presentation to movement onset) were faster for the habitual condition compared to the non-habitual condition. Thumb-toward grips seemed to be the preferred movement choice for this task, as can be seen in the behavioral data for free choice grips. This might have explained the faster reaction times, as participants would most likely have chosen thumb-towards grips themselves, if the grips would not have been specified. The faster reaction times in the habitual condition further indicated that actions executed in the habitual mode require less cognitive effort. Reaction times did not differ significantly between free grip choice and specified grip choice conditions. This was in line with previous findings from our lab (Westerholz et al., unpublished data). The final effect of an action sequence seemed to be more important for action planning than initial grips. As the final effect of the action sequence did not change depending on whether the grip was specified or not, planning processes taking place before the action were not influenced essentially.

Reach times (from movement onset to rotation start) were faster for habitual compared to non-habitual grips. The differences could have been explained with more experience for the habitual action, as less decision making has to be done after action initiation compared to the non-habitual grips. Reach times for the free grip choice condition were faster compared to the specified grip choice condition. This result was in line with previous findings [17] (Westerholz et al., unpublished data). Reach times for the free grip choice condition could have been faster because actions based on self regulation seemed to be more flexible and modifiable than actions based on an instructed plan [23], which made online planning and motor implementation processes more effortless and, thus, faster.

Rotation times (from rotation start to rotation end) did not differ significantly between habitual and non-habitual conditions. This finding came as a surprise, as we expected the homing in phase to be faster for habitual grips. The behavioral results of the free grip choice condition, which show a strong tendency to use thumb-toward grips, suggested that a thumb-toward grip offers participants more control and precision at or near the target position [8]. Maybe this advantage in control did not necessarily provide a temporal advantage as well. Rotation times were faster for free grip choices compared to specified grip choices. As participants were able to choose the optimal strategy, end-state comfort and/or thumb-toward, for every target position in the free grip choice condition, they executed their preferred homing in movement all the time, which were probably the fastest movements as well. In the specified grip choice condition, participants had to execute preferred and undesired homing in movements, which could have slowed down their average rotation times.

Consistent with the hypothesis that the neural processes for action execution would differ between habitual and non-habitual conditions, we observed differential frontal activity between both conditions. The differential activity occurred between −600 and 200 ms time-locked to reaching the final rotation goal. In the time window from −600 to 200 ms there was a negativity for the non-habitual trials compared to the habitual trials in the AL- and AR-ROIs. This seemed to fit with the assumption that the homing in phase was more difficult with the thumb held away from the pointer than towards the pointer [8]. It also fitted with the assumption that frontal areas were involved in supporting final action goals and played a role in planning and control of sequential actions [16].

Note that participants executed the same rotation movements in both conditions. Thus, the movements themselves cannot explain the effect. Participants also finished rotations with the same posture in both conditions. Thus, the final posture cannot explain the effect per se. What differed between conditions was the combination of the movement and final posture. In other words, the difference was whether participants were homing in on the target location with their thumb toward the pointer or with their thumb away from the pointer. The cerebral activity could have represented this difference. The negativity for the non-habitual condition could have been due to more effortful control processes near the target location. Online planning and control processes in the non-habitual condition could have been more effortful because of less experience with thumb-away grips especially in conjunction with the critical part of the movement, as we observed no other effects during the action sequence.

One might wonder, if another explanation for the effect could have been a systematic eye-movement artifact. Participants could have focused their gaze differently during the homing in phase when grasping thumb towards compared to grasping thumb away. Rosenbaum et al. [8] hypothesized that grasping thumb toward might be perceptually advantageous for such a task. Eye movements could have provided better visibility of the pointer close to the target position. However, as we instructed participants to keep their gaze fixed on the screen throughout the movement and we corrected for ocular artifacts using the procedure by Gratton et al. [26], it was highly unlikely that eye-movements caused the observed effect.

To our surprise, we observed no significant effect in the time range from −900 to 0 ms time-locked to grasping for the non-habitual condition compared to the habitual condition. Reaction and reach time differences between the non-habitual and the habitual condition suggested planning and control processes to be easier, and thus faster, for the habitual condition. We expected such differences to appear in the neurophysiological data, based on previous findings [16] (Westerholz et al., unpublished data). These previous experiments required participants to lift an object and place it down at a target location. In contrast, the present experiment did not involve a transport phase. The handle was connected to a disk and had to be grasped and rotated, its orientation changed but its location did not. Maybe the additional transport phase in previous studies [16] (Westerholz et al., unpublished data) caused planning and control processes on a neural level, which do not occur for a rotation movement. Planning and control of the grip might require more precision for an action sequence that involves a transport phase, in order to pick up the object carefully and not to drop it. These suggestions are in line with the functional distinction of transport phase and grasping [28].

As expected, we found no significant difference between the neural processes for action execution in free grip choice and specified grip choice trials. This result was in line with previous findings (Westerholz et al., unpublished data), which showed different cerebral activity between self-regulated and instructed conditions only when the action effect was manipulated. As we did not manipulate the action effect between conditions, no significant difference between the neural processes for both conditions was observed. In accordance with previous suggestions [6], this result may indicate that planning and execution of a movement sequence were not based on initial grips but on the final action effect, which, in this case, was also the moment that required most control. Specifying the action effect thus influenced planning processes for the action, while specifying the grip had no major influence for planning processes of the action, as the desired action effect could be reached regardless of which grip is used. The importance of action effects compared to initial grips has further been demonstrated in a study by Van Elk et al. [29] whose participants were faster in judging the correctness of an action, when asked to focus on the goal of the action than when instructed to attend to the grip of the action. Our findings further support the idea that achieving optimal required control where it is most needed, is of crucial importance for action planning and execution.

For future research it might be of interest to focus on the investigation of the end-state comfort effect. Participants in our experiment showed a strong tendency toward the thumb-toward bias, while the end-state comfort appeared less often than reported before [8]. Participants showed the end-state comfort effect in about 50% of the trial for the most uncomfortable end-posture. A comparison of these thumb-toward and thumb-away grips in the free grip condition might help us to better understand anticipatory grip planning and execution processes. We did not compare any data for only one end-posture because of the reduced number of trials. A future study might focus on specific end positions to collect data for a comparison of comfortable and uncomfortable free grip choices. Another interesting idea for future studies would be a comparison between habitual specified vs. habitual non-specified grips, and non-habitual specified vs. non-habitual non-specified. This comparison would provide a more detailed account of differences between specified and non-specified grips. It could further demonstrate that the habitual grip type whether specified or not is faster and requires less cognitive effort. Our present dataset did not allow this comparison, as splitting the data did not result in enough trials for each condition to do valid analyses.

In sum, we found that reaction and reach times, as well as ERPs differed between habitual and non-habitual grasping actions, suggesting that actions in the habitual mode require less cognitive processing effort for control demanding parts of an action sequence compared to the non-habitual mode. Differences in neural activity occurred from −600 to 200 ms time-locked to reaching the target location of the rotation task in left and right frontal areas. To our knowledge, this is the first study to differentiate cerebral activity underlying overt goal-related actions executed with a habitual or non-habitual grip. Our results indicated that the planning and execution of goal-related actions were controlled by neural mechanisms which depended on the precision and control requirements of the action in the homing in phase.

## Supporting Information

Table S1 100 ms-time-step-analyses time-locked to rotation onset. F-Values for the 3-way interactions of the ANOVAs with the factors Condition, Front-Back, and Left-Right; significant values in bold face (p<0.05). ROIs and t-values are reported only for significant effects of Condition (thumb toward vs. thumb away; p<0.05) as follow-up analyses for significant 3-way interactions; see also text. On average 30 trials per participant for the thumb toward condition and 29 trials for the thumb away condition entered analyses.
(DOCX)

Table S2 100 ms-time-step-analyses time-locked to rotation onset. F-Values for the 3-way interactions of the ANOVAs with the factors Condition, Front-Back, and Left-Right; significant values in bold face (p<0.05). ROIs and t-values are reported only for significant effects of Condition (free grip vs. specified grip; p<0.05) as follow-up analyses for significant 3-way interactions; see also text. On average 60 trials per participant for the free grip condition and 59 trials for the specified grip condition entered the analyses.
(DOCX)

Table S3 100 ms-time-step-analyses time-locked to rotation end. F-Values for the 3-way interactions of the ANOVAs with the factors Condition, Front-Back, and Left-Right; significant values in bold face (p<0.05). ROIs and t-values are reported only for significant effects of Condition (thumb toward vs. thumb away; p<0.05) as follow-up analyses for significant 3-way interactions; see also text. On average 60 trials per participant for the thumb toward condition and 58 trials for the thumb away condition entered the analyses.
(DOCX)

Table S4 100 ms-time-step-analyses time-locked to rotation end. F-Values for the 3-way interactions of the ANOVAs with the factors Condition, Front-Back, and Left-Right; significant values in bold face (p<0.05). ROIs and t-values are reported only for significant effects of Condition (free grip vs. specified grip; p<0.05) as follow-up analyses for significant 3-way interactions; see also text. On average 60 trials per participant for the free grip condition and 58 trials for the specified grip condition entered the analyses.
(DOCX)
