# Supplementary material for: Habitual vs Non-Habitual Manual Actions: An ERP Study on Overt Movement Execution
Source: PLoS One. 2014 Apr 1;9(4):e93116. doi: 10.1371/journal.pone.0093116 (PMC3972190; doi:10.1371/journal.pone.0093116)
Supplement: Table S1 — 100 ms-time-step-analyses time-locked to rotation onset. F-Values for the 3-way interactions of the ANOVAs with the factors Condition, Front-Back, and Left-Right; significant values in bold face (p<0.05). ROIs and t-values are reported only for significant effects of Condition (thumb toward vs. thumb away; p<0.05) as follow-up analyses for significant 3-way interactions; see also text. On average 30 trials per participant for the thumb toward condition and 29 trials for the thumb away condition entered analyses. (DOCX) [file pone.0093116.s001.docx]

| Time window | -2100  -2000 | -2000  -1900 | -1900  -1800 | -1800  -1700 | -1700  -1600 | -1600  -1500 | -1500  -1400 | -1400  -1300 | ...  ... |
| --- | --- | --- | --- | --- | --- | --- | --- | --- | --- |
| F(4,108) | 1.32 | 1.42 | 2.19 | 2.47 | 1.78 | 1.67 | 1.46 | 1.12 |  |
| t(27) |  |  |  |  |  |  |  |  |  |
| Time window | -1300  -1200 | -1200  -1100 | -1100  -1000 | -1000  -900 | -900  -800 | -800  -700 | -700  -600 | -600  -500 | ...  ... |
| F(4,108) | 0.98 | 1.05 | 0.87 | 0.88 | 0.61 | 0.69 | 0.67 | 0.81 |  |
| t(27) |  |  |  |  |  |  |  |  |  |
| Time window | -500  -400 | -400  -300 | -300  -200 | -200  -100 | -100  0 | 0  100 | 100  200 | 200  300 | ...  ... |
| F(4,108) | 0.97 | 1.07 | 1.35 | 1.37 | 1.48 | 1.75 | 2.01 | 2.17 |  |
| t(27) |  |  |  |  |  |  |  |  |  |
| Time window | 300  400 | 400  500 | 500  600 | 600  700 | 700  800 | 800  900 | 900  1000 | 1000  1100 | 1100  1200 |
| F(4,108) | 2.24 | 2.24 | 2.30 | 2.40 | 2.30 | 2.42 | 2.45 | 2.30 | 2.17 |
| t(27) |  |  |  |  |  |  |  |  |  |
