# Supplementary material for: Habitual vs Non-Habitual Manual Actions: An ERP Study on Overt Movement Execution
Source: PLoS One. 2014 Apr 1;9(4):e93116. doi: 10.1371/journal.pone.0093116 (PMC3972190; doi:10.1371/journal.pone.0093116)
Supplement: Table S2 — 100 ms-time-step-analyses time-locked to rotation onset. F-Values for the 3-way interactions of the ANOVAs with the factors Condition, Front-Back, and Left-Right; significant values in bold face (p<0.05). ROIs and t-values are reported only for significant effects of Condition (free grip vs. specified grip; p<0.05) as follow-up analyses for significant 3-way interactions; see also text. On average 60 trials per participant for the free grip condition and 59 trials for the specified grip condition entered the analyses. (DOCX) [file pone.0093116.s002.docx]

| Time window | -2100  -2000 | -2000  -1900 | -1900  -1800 | -1800  -1700 | -1700  -1600 | -1600  -1500 | -1500  -1400 | -1400  -1300 | ...  ... |
| --- | --- | --- | --- | --- | --- | --- | --- | --- | --- |
| F(4,108) | 0.35 | 0.19 | 0.06 | 0.18 | 0.19 | 0.20 | 0.66 | 0.30 |  |
| t(27) |  |  |  |  |  |  |  |  |  |
| Time window | -1300  -1200 | -1200  -1100 | -1100  -1000 | -1000  -900 | -900  -800 | -800  -700 | -700  -600 | -600  -500 | ...  ... |
| F(4,108) | 0.33 | 0.27 | 0.34 | 0.29 | 0.18 | 0.27 | 0.33 | 0.52 |  |
| t(27) |  |  |  |  |  |  |  |  |  |
| Time window | -500  -400 | -400  -300 | -300  -200 | -200  -100 | -100  0 | 0  100 | 100  200 | 200  300 | ...  ... |
| F(4,108) | 0.72 | 1.00 | 1.13 | 1.10 | 0.94 | 0.89 | 0.82 | 0.47 |  |
| t(27) |  |  |  |  |  |  |  |  |  |
| Time window | 300  400 | 400  500 | 500  600 | 600  700 | 700  800 | 800  900 | 900  1000 | 1000  1100 | 1100  1200 |
| F(4,108) | 0.50 | 0.53 | 0.50 | 0.39 | 0.63 | 0.67 | 0.73 | 0.94 | 0.91 |
| t(27) |  |  |  |  |  |  |  |  |  |
