# Supplementary material for: Habitual vs Non-Habitual Manual Actions: An ERP Study on Overt Movement Execution
Source: PLoS One. 2014 Apr 1;9(4):e93116. doi: 10.1371/journal.pone.0093116 (PMC3972190; doi:10.1371/journal.pone.0093116)
Supplement: Table S3 — 100 ms-time-step-analyses time-locked to rotation end. F-Values for the 3-way interactions of the ANOVAs with the factors Condition, Front-Back, and Left-Right; significant values in bold face (p<0.05). ROIs and t-values are reported only for significant effects of Condition (thumb toward vs. thumb away; p<0.05) as follow-up analyses for significant 3-way interactions; see also text. On average 60 trials per participant for the thumb toward condition and 58 trials for the thumb away condition entered the analyses. (DOCX) [file pone.0093116.s003.docx]

| Time window | -3100  -3000 | -3000  -2900 | -2900  -2800 | -2800  -2700 | -2700  -2600 | -2600  -2500 | -2500  -2400 | -2400  -2300 | -2300  -2200 |
| --- | --- | --- | --- | --- | --- | --- | --- | --- | --- |
| F(4,108) | 0.07 | 0.79 | 0.74 | 0.93 | 0.71 | 0.56 | 0.56 | 0.83 | 0.66 |
| t(27) |  |  |  |  |  |  |  |  |  |
| Time window | -2200  -2100 | -2100  -2000 | -2000  -1900 | -1900  -1800 | -1800  -1700 | -1700  -1600 | -1600  -1500 | -1500  -1400 | -1400  -1300 |
| F(4,108) | 0.92 | 1.11 | 0.97 | 0.99 | 0.73 | 0.83 | 0.84 | 0.73 | 0.86 |
| t(27) |  |  |  |  |  |  |  |  |  |
| Time window | -1300  -1200 | -1200  -1100 | -1100  -1000 | -1000  -900 | -900  -800 | -800  -700 | -700  -600 | -600  -500 | -500  -400 |
| F(4,108) | 1.01 | 1.01 | 1.12 | 1.84 | 2.08 | 2.00 | 2.69 | **3.16** | **3.31** |
| t(27) |  |  |  |  |  |  |  | 2.07 AL  2.19 AR | 2.14 AL  2.11 AR |
| Time window | -400  -300 | -300  -200 | -200  -100 | -100  0 | 0  100 | 100  200 | 200  300 |  |  |
| F(4,108) | **3.36** | **3.50** | **3.70** | **4.20** | **4.31** | **3.94** | **4.10** |  |  |
| t(27) | 2.40 AL  2.30 AR | 2.44 AL  2.29 AR | 2.44 AL  2.19 AR | 2.43 AL  2.08 AR | 2.24 AL | 2.08 AL |  |  |  |
